# Evaluation of the SsIR/NIE recombinant antigen ELISA for the follow up of patients infected by *Strongyloides stercoralis*: a diagnostic study

**DOI:** 10.1017/S0031182024000027

**Published:** 2024-03

**Authors:** Marco Prato, Francesca Tamarozzi, Stefano Tais, Eleonora Rizzi, Cristina Mazzi, Dora Buonfrate

**Affiliations:** Department of Infectious Tropical Diseases and Microbiology, IRCCS Sacro Cuore Don Calabria Hospital, Verona, Negrar di Valpolicella, Italy

**Keywords:** ELISA, follow up, serology, Strongyloides, strongyloidiasis

## Abstract

Some serology assays demonstrated useful for post-treatment monitoring of *Strongyloides stercoralis* infection. Serology frequently has low specificity, which might be improved by the use of recombinant antigens. The Strongy Detect ELISA is based on 2 recombinant antigens (SsIR and NIE) and proved good accuracy. Aim of this study was to evaluate the performance of this test for the post-treatment monitoring of strongyloidiasis. We tested 38 paired sera, with matched fecal tests results, stored in our biobank and originating from a randomized controlled trial. At baseline, all patients tested positive for at least 1 fecal assay among PCR, direct stool microscopy and agar plate culture. Patients were re-tested with both serology and fecal assays 12 months after treatment. Primary outcome was the relative reduction in optical density (OD) between baseline and follow up. We observed that about 95% samples showed a reduction between pre and post-treatment OD, with a median relative reduction of 93.9% (IQR 77.3%–98.1%). In conclusion, the test proved reliable for post-treatment monitoring. However, some technical issues, including that the threshold for positivity has not be predefined, and that a substantial number of samples showed overflow signals, need to be fixed to permit use in routine practice.

## Graphical abstract

**Figure:**
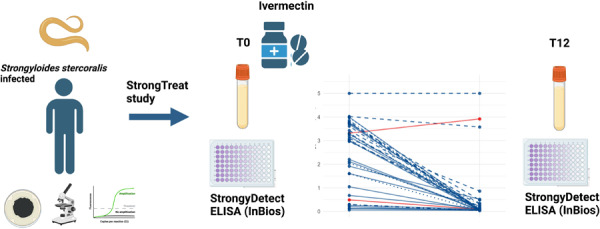

## Introduction

*Strongyloides stercoralis* is a soil-transmitted helminth which can cause relevant morbidity in affected people, and a life-threatening syndrome in immunocompromised individuals (Buonfrate *et al*., 2023). The global strongyloidiasis prevalence in 2017 was estimated to be 8.1% (around 600 million people), mainly distributed in tropical and sub-tropical areas of South-East Asia, Africa and Western Pacific (Buonfrate *et al*., 2020). The infection is acquired through direct contact with soil contaminated with infective filariform larvae (iL3), which can actively penetrate the host skin. The larvae moult while migrating through the human tissues, and eventually mature into adult parthenogenic female worms in the small intestine, where they produce eggs. The latter hatch immediately, releasing rhabditoid larvae (L1), which are either excreted with feces, contaminating the soil in case of open defecation, or moult into iL3 when still in the bowel. These iL3 can penetrate the perianal skin or the mucosa of the rectum, leading to the auto-infective cycle of this parasite, thus to chronic infection of the host (Nutman, 2017).

Release of larvae in the feces is intermittent and variable in terms of number of parasites over time, hence direct parasitological diagnosis has low sensitivity (Krolewiecki *et al*., 2013). Other fecal-based tests, such as agar plate culture (APC), Baermann method and also real-time PCR, demonstrated better but still unsatisfactory sensitivity (Buonfrate *et al*., 2022). Currently, serology is considered the diagnostic method with the highest sensitivity, though specificity can be affected by cross-reactions. Moreover, accuracy varies widely between different assays (van Doorn *et al*., 2007; Ramanathan *et al*., 2008; Krolewiecki *et al*., 2010; Norsyahida *et al*., 2013; Bisoffi *et al*., 2014). Recently, a novel ELISA assay based on 2 recombinant antigens (Strongy Detect, InBios International, Inc., Seattle, WA, USA) was implemented and evaluated in 2 retrospective studies (Tamarozzi *et al*., 2021; Sears and Nutman, 2022), one of them carried out in a clinical setting. In both studies, the assay demonstrated good accuracy, with excellent specificity (around 98% in both studies) and good sensitivity (ranging from 78% to 98.6%). More recently, the assay has also been evaluated in a prospective diagnostic study carried out in Ecuador (Tamarozzi *et al*., 2023). In the latter study, the test was performed using dried blood spots collected on filter paper, and sensitivity and specificity were 83.5% (credible intervals [CI] 73.8–91.8) and 91.7% (CI 89.6–93.8), respectively. The different study design (retrospective *vs* prospective), population (*ad hoc* laboratory cohorts, migrants, children living in endemic area), sample collection (frozen sera, dried blood spots), and method of analysis (comparison with fecal tests, latent class analysis) might be the cause of the discrepant results between studies.

In addition to good accuracy for individual diagnosis and screening, an assay should be able to evaluate the response to treatment. Fecal-based tests are not completely reliable for post-treatment monitoring since, due to their low sensitivity, a negative test is not sufficient to exclude the persistence of the infection, especially in case of low larval load.

Previous studies have shown that some sero-assays (both crude antigens and recombinant proteins-based ELISAs) are useful for post-treatment follow up of patients with strongyloidiasis (Loutfy *et al*., 2002; Biggs *et al*., 2009; Buonfrate *et al*., 2015; Kearns *et al*., 2017). It might take a long period of time (sometimes more than 12 months) for seroreversion to occur, but a number of studies demonstrated that a 50% decrease of the optical density (OD) ratio from baseline to follow up can be used as a surrogate marker of cure (Buonfrate *et al*., 2022).

Aim of this study is to evaluate the performance of the InBios Strongy Detect ELISA for the post-treatment follow-up of patients with strongyloidiasis in a clinical context, taking advantage of well-characterized sera originating from a multicentre randomized controlled trial for the treatment of strongyloidiasis (Strong Treat trial) (Buonfrate *et al*., 2019).

Specifically, the primary objective of this study was to evaluate whether post-treatment sera tested with InBios Strongy Detect ELISA show at least a 50% reduction of the antibody titre against *S. stercoralis* compared to baseline.

## Materials and methods

### Study design

This was a retrospective, single-centre diagnostic study performed on sera stored in the ‘Tropica Biobank’ of the Department of Infectious Tropical diseases and Microbiology (DITM) of IRCCS Sacro Cuore Don Calabria hospital, Negrar, Verona, Italy. Results are reported following the STARD (Standards for Reporting Diagnostic Accuracy) checklist (Bossuyt *et al*., 2016).

### Participants

All sera available in the ‘Tropica Biobank’ and originating from patients enrolled in the Strong Treat trial were eligible for the study, conditional to the availability of both the baseline and the 12-month post-treatment follow up sera. All patients deemed eligible for enrolment in the Strong Treat were tested with both serology and at least 1 fecal test between APC, direct microscopy or PCR. According to the inclusion criteria, patients could be enrolled in case of either positive serology at ‘high’ titre [as defined in a previous diagnostic study (Bisoffi *et al*., 2014)], irrespective of the result of the fecal tests, or positive serology at any titre and positivity in at least 1 fecal test (Buonfrate *et al*., 2019). In the present study, we included only sera from cases with 1 or more positive fecal test(s) at baseline. According to the Strong Treat trial, people living in or planning journeys to areas endemic for *S. stercoralis* were excluded, to rule out possible reinfection, which could affect the antibody response and therefore the seroassay results between baseline and follow-up. All participants to the trial were treated with either single or 4 doses of ivermectin 200 *μ*g kg^−1^, depending on the study arm, and multiple doses did not show higher efficacy than the single dose. At follow up, patients were re-tested 6 and 12 months apart with serology and, in case of any positive fecal test at baseline, with either PCR or APC. Cure was defined by negative fecal tests (when performed) and negative or significantly reduced serology (i.e. decrease of 2 titres in in-house immunofluorescence (IFAT) or a 2fold reduction of normalized OD in the case of ELISA) (Buonfrate *et al*., 2019).

### Test methods

The InBios Strongy Detect ELISA (InBios International, Inc., Seattle, WA, USA) is a sandwich-type assay detecting IgG antibodies against *Strongyloides* recombinant antigens NIE (Ravi *et al*., 2002) and SsIR (Ramanathan *et al*., 2008) in serum. Currently, the assay is available as research-use only (RUO). Positive and negative control samples are provided in the kit. The tests were performed and interpreted according to the manufacturer's instructions. No fixed cut-off is provided by the producer, requiring to calculate the cut-off value at every single use. Due to the absence of a control group of negative individuals, here we could not calculate a cut-off. However, here to identify positive samples at baseline we relied on a cut-off value of 0.675 OD calculated in a previous study (Tamarozzi *et al*., 2021) carried out using a different panel of frozen samples stored in the Tropica Biobank. Briefly, samples and controls were diluted 1:100 in the kit's sample buffer and 100 *μ*L added to antigens-coated 96-well plate test wells. After 30 minutes incubation at 37°C and wash, 100 *μ*L HRP-conjugated anti-human-IgG secondary antibody were added, followed by the second incubation of 30 minutes at 37°C. After washing, 100 *μ*L of liquid TMB substrate were added. The reaction was stopped after 10 minutes by adding 50 *μ*L of stop solution, and read by spectrophotometer at 405 nm (ELx800™ Absorbance Microplate Reader BioTek® Instruments, Inc. Winooski, Vermont USA). 32 baseline and 4 follow-up samples showed an OD value over the superior limit of reading (OD ⩾5, from here on referred to as ‘overflow’). In order to attribute a specific OD value to these samples, after a second thawing of the sera, samples were diluted using the provided sample buffer at ratios of 1:800, 1:1600 and 1:3200, before re-testing. For each baseline/follow up pair of sera, the same dilution ratio was applied, in order to compare the pre- and post-treatment OD values. One sample with overflow signal, both at baseline and follow up, could not be re-tested due to the low quantity of remaining serum. The lab staff who performed and recorded the ELISA results were blinded to the results of all tests performed previously and to the classification of the samples in the Strong Treat trial. Each sample was tested in a single run, unless technical problems occurred. Both baseline and follow-up sera were positioned randomly in the plate and tested in the same run. All tests were run between the 18th and 24th January, 2023.

Methods for APC and PCR used in the context of the Strong Treat trial were described previously (Buonfrate *et al*., 2017). Briefly, for APC 10 agar plates are cultured, each 1 with about 2 g of feces produced within the previous 24 hours, mixed with charcoal and incubated at 26°C. Macroscopic and microscopic observation is carried out after 48 h incubation and thereafter daily up to day 5 in case of negativity. For PCR, an automated extractor instrument (MagEX STARlet platform from Hamilton) is used for DNA extraction from about 200 mg feces. Real-time PCR is performed according to Verweij's method (Verweij *et al*., 2009), targeting the small-subunit rRNA gene sequence of *S. stercoralis*. Positive and negative controls are included in all runs, along with the PhHV-1 control DNA amplified with the appropriate primers/probe mix in the same reaction. The reactions, detection and data analysis were performed with the CFX96 detection system (Bio-Rad Laboratories).

### Analysis

Continuous variables were summarized using median and interquartile ranges (IQR), while discrete variables were summarized by absolute and percentage frequencies. At baseline, positive samples were defined as those with OD ⩾0.675. Comparison between baseline and follow-up OD was performed using Wilcoxon Mann Whitney test, to calculate the relative reduction from baseline to follow-up. The alternative hypothesis of the test was that the relative reductions in OD values between baseline and follow-up were more than 50%. *P* < 0.05 was considered significant.

## Results

**Figure 1.**
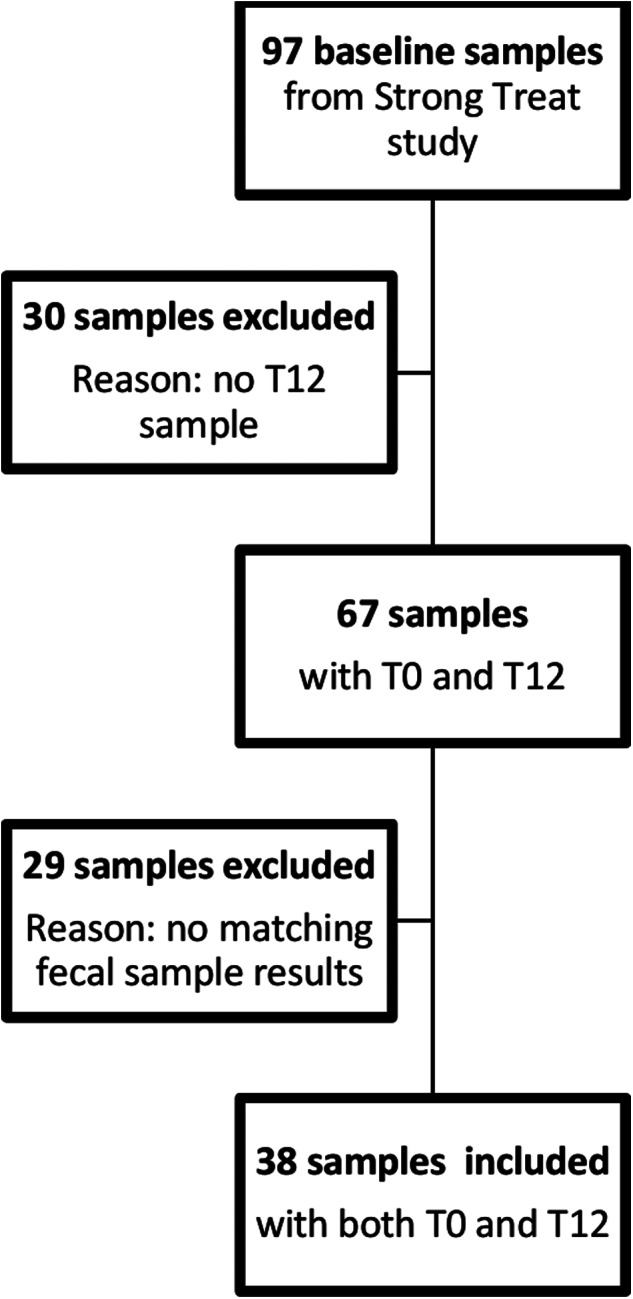
Study flow chart

A total of 97 baseline sera from the Strong Treat trial were available in the Tropica Biobank, for 67 of which was also available a related 12-month follow-up sample. Finally, we included 38 pairs of baseline-follow-up matched samples, which had at least 1 positive stool exam between APC, PCR and direct stool test at baseline. Figure 1 describes the sample selection. Table 1 summarizes the main characteristics of the study population and of the selected samples.

**Table 1.** Main characteristics of the study population and of the selected samples

	*N*	Summary
Age (years)	38	56.2 [IQR 39.9–73.6]
Sex (male)	38	17/38 (44.7%)
Length of follow-up (months)	38	13.6 [IQR 12.9–14.5]
Positive direct stool test at baseline	21	12/21 (57.1%)
Positive APC at baseline	32	30/32 (93.8%)
Positive PCR at baseline	10	8/10 (80.0%)

*N* is the number of non-missing value. Categorical variables are expressed as *n*/*N* (%). Continuous variables are expressed as: median [IQR].

All baseline samples tested positive to the InBios Strongy Detect ELISA.

**Figure 2.**
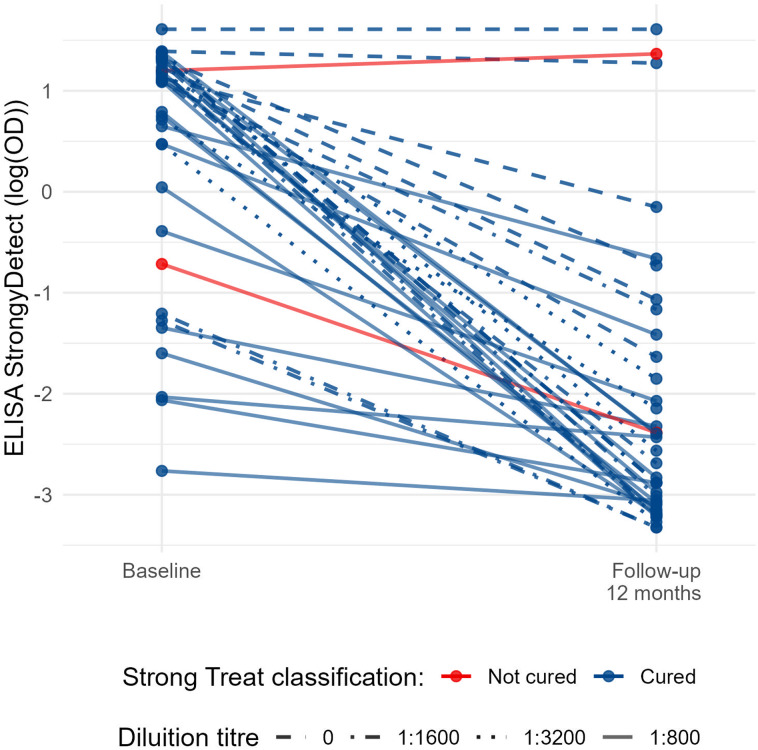
Comparison between baseline and post-treatment optical density (OD). Time is represented on the x-axis; OD values are presented on a log scale on the y-axis. Dilution used for each pair sample is represented by different line patterns, as reported in the graph. All samples originate from the strong treat randomized controlled trial, comparing single and multiple ivermectin doses for treatment of strongyloidiasis: blue lines represent samples from patients who were classified as cured in the Strong Treat, while red lines indicate samples from patients who did not respond to treatment.

As shown in Fig. 2, 94.7% (36/38) of the follow up samples showed an OD reduction compared to baseline, with a median OD relative reduction of 93.9% (IQR 77.3% – 98.1%). 33 out of 38 samples (86.8%) showed at least 50% reduction (i.e. the criterion to define cure) between baseline and follow-up OD values. Of note, 20 out of these 33 samples (52.6% of the whole panel) showed more than 90% OD reduction compared to baseline. Among the samples which did not show an OD reduction of at least 50%, which would suggest persistence of infection, 3 were classified as cured in the Strong Treat trial (hence, results were discrepant between this and the previous study), while 1 sample was classified as ‘not cured’ also in the Strong Treat trial. The latter sample showed an increased OD from baseline to follow up (OD_BL_ = 3.31 *vs* OD_FU_ = 3.92).

## Discussion

This study aimed to evaluate the performance of the Strongy Detect ELISA for the post-treatment follow-up of patients with strongyloidiasis, using samples from a clinical trial on the efficacy of ivermectin (Buonfrate *et al*., 2019). The combined use of the recombinant antigens Ss-NIE and Ss-IR aims at improving specificity and sensitivity of serological assays (Sears and Nutman, 2022; Tamarozzi *et al*., 2022). Improved specificity is particularly relevant for use in endemic areas, where co-infection with other human parasitic nematodes could increase the false positive rate due to cross-reactions (Krolewiecki *et al*., 2010).

Here, we confirmed the good sensitivity of the Strongy Detect ELISA, since all baseline samples (with diagnosis confirmed by positive matched fecal assays results) were positive. We then found a significant decrease in OD values between baseline and 12-month post-treatment sera, suggesting that this ELISA assay might be used for monitoring the treatment response in people with strongyloidiasis. One sample did not show any decrement in antibody response, in line with the results from the Strong Treat trial, hence suggesting persistence of infection in that participant. For 3 participants, the baseline OD did not halve after 12 months from treatment, but still decreased. This might be due to the need for a longer period of follow up to observe OD reduction meeting the halving criterion for cure, possibly due to the high OD value at baseline (as it often occurs also with other assays) (Krolewiecki *et al*., 2010; Buonfrate *et al*., 2015). Along with normal eosinophil count, clearance of symptoms (if any present before treatment) and negative fecal tests (if available), the OD decline, which can be observed in a shorter period compared to seroreversion, can be useful to assess cure, as shown previously (Buonfrate *et al*., 2019). Unfortunately, in this study it is not possible to comment on the rate of seroreversion, because the majority of samples had to be variably diluted, making the use of the cut-off defined on undiluted sera not appropriate.

Our main results are in line with a previous validation study of the Strongy Detect assay, where 35 pre- and post-treatment samples were selected and analysed (Sears and Nutman, 2022) That study showed an overall significant reduction of the antibody levels post-treatment, with seroreversion in a few samples only (Sears and Nutman, 2022). However, the authors did not report the average OD values at baseline and follow-up, nor the number of samples that seroreverted, limiting the comparison between the 2 studies.

This study has several limitations, including the small number of samples. However, our data add up to the previous ones, that were also carried out on a small sample size, so that more evidence can be available. Moreover, we could not calculate a cut-off for positivity, since suitable negative controls were not available. However, for the classification of sera at baseline we relied on the cut-off obtained from the analysis of a previous study carried out on a different cohort obtained in a similar context; applying this cut-off, all samples examined in this study would result positive at baseline. The study was also limited by a technical issue due to the high number of samples which showed overflow signal, requiring a dilution step before the procedure.

The absence of a predefined cut-off value and the overflow results question the applicability of this assay in practice, where serology is often used as screening test. Taking into consideration both the robustness and scalability of the recombinant antigens but also the lack of a provided fixed cut-off value or the necessity to add some pre-dilution steps before using the assay, it would be worth revising and improving this test both for individual diagnosis and for control programs in endemic settings. Should the manufacturer solve the technical issues, there is some preliminary evidence supporting the role of this assay in screening (Tamarozzi *et al*., 2023) and post-treatment monitoring, encouraging further studies on this assay, once the technical problems are solved.

## Conclusions

The Strongy Detect ELISA detected a decline in antibody levels 12 months after treatment in most samples tested, thus this assay might be a reliable method for post-treatment monitoring. However, the lack of a defined cut-off value from the manufacturer and technical issues due to the excessive proportion of samples showing overflow results when tested following manufacturer's instruction dilutions, currently limit its application both in a clinical setting and in the context of control programmes in endemic areas.

## Data Availability

The raw data of this study are available in Zenodo: https://doi.org/10.5281/zenodo.10554353.
